# Serum 25-Hydroxyvitamin D Levels and Disease Activity in Patients with Systemic Lupus Erythematosus: An Exploratory Study in Western Mexico

**DOI:** 10.3390/diseases12120319

**Published:** 2024-12-08

**Authors:** Daniela Deossa-Piedrahita, Berenice Vicente-Hernández, Sol Ramírez-Ochoa, Mauricio Alfredo Ambriz-Alarcón, Gabino Cervantes-Pérez, Gabino Cervantes-Guevara, Alejandro González-Ojeda, Clotilde Fuentes-Orozco, Francisco Javier Hernández-Mora, Luis Asdruval Zepeda-Gutiérrez, Jorge Isaac Michel-González, Janet Cristina Vázquez-Beltrán, Enrique Cervantes-Pérez

**Affiliations:** 1 Department of Internal Medicine, Hospital Civil de Guadalajara Fray Antonio Alcalde, Guadalajara 44280, JAL, Mexico; daniosa15@hotmail.com (D.D.-P.); bvicente@hcg.gob.mx (B.V.-H.); ramirez_ochoa_sol@hotmail.com (S.R.-O.); gabino-1994@hotmail.com (G.C.-P.); luis-zent@hotmail.com (L.A.Z.-G.); meidevilll@gmail.com (J.I.M.-G.); 2 División de Servicios Intermedios, Hospital Civil de Guadalajara Fray Antonio Alcalde, Guadalajara 44280, JAL, Mexico; mau_ambriz@hotmail.com; 3 Department of Gastroenterology, Hospital Civil de Guadalajara Fray Antonio Alcalde, Guadalajara 44280, JAL, Mexico; gabino_guevera@hotmail.com; 4 Department of Welfare and Sustainable Development, Centro Universitario del Norte, Universidad de Guadalajara, Guadalajara 46200, JAL, Mexico; 5 Biomedical Research Unit 02, Hospital de Especialidades, Centro Médico Nacional de Occidente, Guadalajara 44350, JAL, Mexico; avygail5@gmail.com (A.G.-O.); clotilde.fuentes@gmail.com (C.F.-O.); 6 Department of Human Reproduction, Growth and Child Development, Health Sciences University Center, Universidad de Guadalajara, Guadalajara 44280, JAL, Mexico; frank.gine@gmail.com; 7 Department of Obstetrics, Hospital Civil de Guadalajara Fray Antonio Alcalde, Guadalajara 44280, JAL, Mexico; 8 Department of Medical Sciences, Instituto Técnológico y de Estudios Superiores de Monterrey, Campus Guadalajara, Guadalajara 45201, JAL, Mexico; 9 School of Medicine, Instituto Politécnico Nacional, Mexico City 11340, CDMX, Mexico; janet.cris.beltran@gmail.com; 10 Department of Philosophical, Methodological and Instrumental Disciplines, Centro Universitario de Ciencias de la Salud, Universidad de Guadalajara, Guadalajara 44340, JAL, Mexico; 11 Departmento de Clínicas, Centro Universitario de Tlajomulco, Universidad de Guadalajara, Guadalajara 45641, JAL, Mexico

**Keywords:** 25-hydroxyvitamin D, disease activity, systemic lupus erythematosus, vitamin D

## Abstract

Background and objectives: The correlation between diminished 25-hydroxyvitamin D (25-(OH)D) concentrations and heightened disease activity in systemic lupus erythematosus (SLE) patients remains contentious, as clinical studies have yielded conflicting outcomes—some propose a potential link, while others assert no relationship exists. Nonetheless, all studies report a significant prevalence of low 25-(OH)D levels among SLE patients. This study aimed to assess the frequency of low serum levels of 25-(OH)D in Mexican patients with SLE and to evaluate the correlation between 25-(OH)D deficiency or insufficiency and disease activity levels. Materials and Methods: This retrospective analysis comprised patients admitted to our hospital from November 2022 to October 2023, diagnosed with SLE, and had their serum 25-(OH)D levels tested upon admission. The frequency of low levels of 25-(OH)D was assessed, and clinical and demographic data were gathered to examine potential causes linked to 25-(OH)D deficiency or insufficiency. Results: A total of 61 patients were included, and 87% (n = 53) had low serum 25-(OH)D levels. Patients with 25-(OH)D deficiency (n = 21) were significantly younger (mean 23 vs. 39 years, *p* = 0.04) and had higher protein levels in 24 h urine protein (1.8 vs. 1.1 g/24 h, *p* = 0.006) than patients who presented only 25-(OH)D insufficiency, without significant differences in other indicators of disease activity. Conclusions: In this investigation, patients with SLE exhibited a high frequency of low serum levels of 25-(OH)D, consistent with existing literature; however, no significant correlations were identified between 25-(OH)D levels and indicators of disease activity. These findings require validation in subsequent research.

## 1. Introduction

In recent decades, the management of systemic lupus erythematosus (SLE) has tended to shift from hydroxychloroquine, systemic glucocorticosteroids, and traditional immunosuppressive medications to biological therapies [1]. Adjuvant treatments and nonpharmacological interventions are essential components in the management of patients with SLE. Dietary modifications due to the high prevalence of intestinal microbiome dysbiosis in this patient population, photoprotection to mitigate skin manifestations associated with ultraviolet exposure in patients with SLE and cutaneous lupus erythematosus (CLE) [1,2,3], and dietary supplementation with 25-hydroxyvitamin D (25-(OH)D) [4] are particularly noteworthy. The latter has gained significance due to the high prevalence of low serum levels of 25-(OH)D in patients with SLE, which has been linked to increased disease activity in certain studies. Additionally, dietary supplementation of 25-(OH)D has been reported to alleviate symptoms such as fatigue and enhance physical activity levels in SLE patients. However, the findings remain inconclusive and have primarily emerged from small-scale studies [4,5,6,7].

Various hypotheses have been suggested regarding the relationship between 25-(OH)D and the pathophysiology of SLE, with some positing that the imbalance in immunomodulation may be affected by 25-(OH)D levels [8,9,10,11].

The association between low 25-(OH)D levels and heightened disease activity in patients with SLE is disputed, as numerous clinical investigations yielded contradictory results [12]. Certain studies proposed a possible relationship [13,14,15,16], while others demonstrated no correlation between 25-(OH)D levels and disease activity [17]. Nonetheless, a significant prevalence of low 25-(OH)D levels has been consistently observed in SLE patients, leading to recommendations for dietary supplementation with 25-(OH)D due to its potential benefits and high prevalence in this population [18,19,20].

Investigations on vitamin D supplementation in individuals with SLE yielded incongruous results. Certain reviews suggested a beneficial influence of vitamin D supplementation on disease activity, but others revealed no effect [5,18,20,21,22,23].

The most recent data on the prevalence of 25-(OH)D deficiency in the general population in Mexico were from the National Health and Nutrition Survey (ENSANUT), conducted in 2022 [24]. For the purposes of the survey, 25-(OH)D deficiency was defined as serum 25OH 25-(OH)D levels < 20 ng/mL. The prevalence of 25-(OH)D deficiency in preschool children (1–4 years of age) was 4.7%, with an average of 27.4 ng/mL. In school-aged children (5–11 years of age), the prevalence was 23.3%, with an average of 23.8 ng/mL. In non-pregnant women aged 12–49 years, the prevalence was 37.7%, with an average of 22 ng/mL. In children aged 1–4 years, the prevalence was 4.7%, with a mean of 27.4 ng/mL. In children aged 5–11 years, the prevalence was 23.3%, with an average of 23.8 ng/mL. In non-pregnant women aged 12–49 years, the prevalence was 37.7%, with an average of 22 ng/mL. The prevalence of deficiency was higher in all population groups studied among subjects residing in urban areas. In the data reported in ENSANUT in 2006 [25], it was found that the average serum level of 25-(OH)D in Mexican adults over 20 years of age was 38.8 ng/mL, with a frequency of deficiency of less than 8. The prevalence of insufficiency (serum levels < 20 ng/mL) was 10% overall, while the prevalence of inadequacy (serum levels < 30 ng/mL) was 20%.

The study by García-Carrasco et al. on Mexican patients with SLE indicated a mean 25(OH)D value of 19.7 ± 6.3 ng/mL; 89% of the SLE patients exhibited 25-(OH)D insufficiency (levels below 30 ng/mL), while 2.9% showed deficiency. Consequently, 91.9% of the participants demonstrated reduced 25-(OH)D levels [17].

A global consensus on the ideal serum levels of 25-(OH)D is lacking. The definitions of deficiency, insufficiency, and inadequacy differ significantly and are substantiated by many experimental findings. In numerous clinical studies, serum levels below 30 ng/mL are considered indicative of vitamin D insufficiency because increasing serum 25-(OH)D levels from 20 ng/mL to 32 ng/mL results in an average enhancement of intestinal calcium transport by 45 to 65% in women (no experimental data exists for men). Additionally, there is an inverse relationship between calcium levels and parathyroid hormone as well as serum 25-(OH)D levels, which diminishes at levels between 30 and 45 ng/mL, where parathyroid hormone levels begin to decline from their nadir [26]. A 25-(OH)D level below 10 or 12 ng/mL is considered a severe deficiency (or simply deficiency) of 25-(OH)D [27], as it is associated with an increased frequency of osteomalacia and nutritional rickets. In its Dietary Reference Intakes for Calcium and 25-(OH)D, the Institute of Medicine recommends a cutoff point for deficiency of <20 ng/mL. However, this recommendation is intended to establish a threshold for initiating dietary supplementation rather than addressing immunological considerations (such as those associated with SLE) [28].

A consensus has not yet been reached regarding the causal relationship of 25-(OH)D deficiency and SLE; that is, it has not yet been determined whether the observed alterations are a consequence or one of the causal factors of SLE [26,29,30,31,32,33].

This study aimed to ascertain the frequency of low serum levels of 25-(OH)D in Mexican patients with SLE at a hospital in western Mexico and to evaluate the correlation between 25-(OH)D deficiency or insufficiency and disease activity levels assessed through various clinical indicators.

## 2. Materials and Methods

This retrospective cross-sectional study encompassed patients of both genders, aged 18 to 60, who were admitted to the internal medicine department of “Hospital Civil de Guadalajara Fray Antonio Alcalde” from 1 November 2022 to 31 October 2023. Eligible patients were diagnosed with SLE according to the criteria established by the American College of Rheumatology/Systemic Lupus International Collaborating Clinics (ACR/SLICC) [34,35] and had serum 25-(OH)D levels assessed upon admission. The exclusion criteria included pregnancy, pre-existing liver illness, a history of malignancy, chronic kidney disease, hyperparathyroidism, and the use of anticonvulsants, theophylline, or antituberculosis drugs.

Data were extracted from the patients’ electronic medical records, encompassing blood counts, blood chemistry, complement C3 and C4 levels, erythrocyte sedimentation rate (ESR), C-reactive protein (CRP), serum 25-(OH)D levels, pertinent clinical history, demographic information, and systemic lupus erythematosus disease activity scores (SLEDAI-2K and mex-SLEDAI) [36]. All data were individually recorded in an Excel spreadsheet (Microsoft Office Excel 2016, Microsoft Corporation, Redmond, WA, USA) for subsequent statistical analysis.

Serum 25-(OH)D concentrations were quantified via a chemiluminescence immunoassay (Human 25-Hydroxy Vitamin D CLIA kit, Epitope Biotechnology, Jiaxing Zhejiang, China) for the precise measurement of 25-(OH)D. During the initial incubation phase, 25-(OH)D was released from its binding protein and subsequently attached to a particular antibody on a solid phase. Subsequent to a 10 min interval, a tracer (25-(OH)D conjugated to an isoluminol derivative) was introduced, followed by a further 10 min incubation. Unbound material was eliminated via a washing cycle, and initiator reagents were introduced to activate the chemiluminescent reaction. The detected light signal, quantified in relative light units by a photomultiplier, exhibited an inverse correlation with the concentration of 25-(OH)D present. Calibration was performed according to the manufacturer’s recommendations and supervised by the quality control department of the hospital’s clinical laboratory. 25-(OH)D insufficiency was defined as serum levels of 25-(OH)D below 30 ng/mL to 10 ng/mL ng/mL, and deficiency as <10 ng/mL (9 ng/mL or below) [26,27,37].

Statistical analyses were performed utilizing GraphPad Prism software (version 9.3.1.471, GraphPad by Dotmatics, Boston, MA, USA). Descriptive statistics for qualitative variables were presented as absolute and relative frequencies, expressed in percentages and proportions. Measures of central tendency and dispersion for quantitative variables were computed, including the mean, median, standard deviation, and interquartile range.

Chi-square tests were employed in inferential statistics to compare the proportions of dichotomous categorical variables in 2 × 2 contingency tables. Normality for quantitative variables was evaluated using the D’Agostino–Pearson and Kolmogorov–Smirnov tests, given that the sample size surpassed 50 data points. Comparisons between unpaired groups were conducted using the Student’s *t*-test for nonparametric data, with medians compared where necessary. A *p*-value of less than 0.05 was deemed statistically significant, accompanied by a 95% confidence range.

To evaluate the relationship between serum 25-(OH)D levels and various disease activity indicators—including serum levels of complement C3, C4, SLEDAI-2K, mex-SLEDAI, and statistically significant laboratory variables—Pearson correlation coefficients (r) and simple linear regression analyses were performed for each independent variable, with statistical significance established at *p* < 0.05.

The research was executed in compliance with the Declaration of Helsinki and Good Clinical Practice standards. The Clinical Research and Bioethics Committee of Hospital Civil de Guadalajara “Fray Antonio Alcalde” sanctioned the study (CEI-13/24), and the necessity for informed consent was exempted owing to the retrospective character of the research.

## 3. Results

Our study comprised 61 patients diagnosed with SLE who fulfilled the selection criteria. Among the included patients, 87% (n = 53) were women, 13% (n = 8) were men, and the mean age was 35 years. The proportion of patients with low 25-(OH)D levels was 87% (n = 53), 40% (n = 21) presented 25-(OH)D deficiency, 60% (n = 32) presented 25-(OH)D insufficiency, and the average 25-(OH)D level was 16.8 ng/mL.

Table 1 presents the demographic characteristics and significant clinical history of patients exhibiting low levels of 25-(OH)D. Patients were categorized into two groups: those with 25-(OH)D deficiency (serum levels < 10 ng/mL) and those with 25-(OH)D insufficiency (serum levels between 10 and 29 ng/mL).

Table 2 presents the laboratory findings and clinical attributes of patients with 25-(OH)D deficiency and insufficiency. Patients with 25-(OH)D deficiency exhibited a median SLEDAI-2K score of 12 (interquartile range (IQR) = 11), whereas those with insufficiency had a median score of 9 (IQR = 13), indicating no statistically significant difference (*p* = 0.22). Patients exhibiting vitamin D deficiency, both in the low-moderate SLEDAI-2K score group and the severe group, demonstrated a significantly elevated median total score compared to those with vitamin D insufficiency. Specifically, the median low-moderate SLEDAI-2K score was 11 (IQR = 4) versus 7 (IQR = 4), *p* = 0.003, and the median severe SLEDAI-2K score was 26 (IQR = 12) versus 20 (IQR = 4), *p* = 0.03.

**Figure 1:**
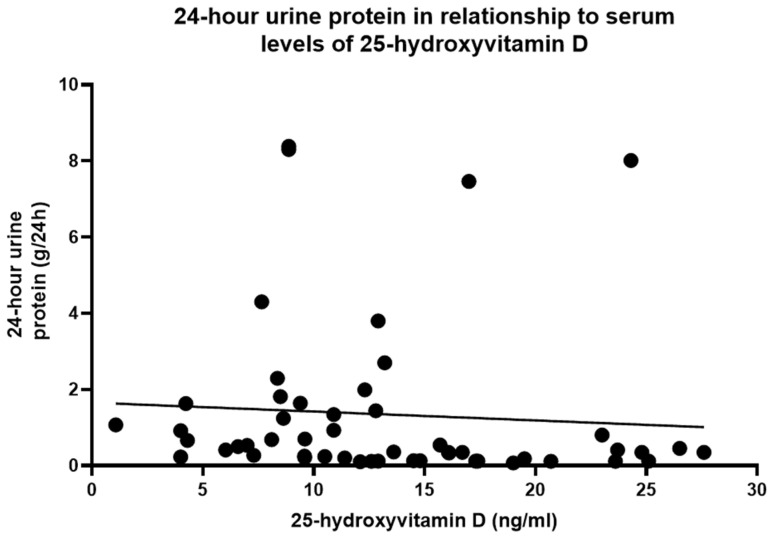
The relationship of 24 h urine protein with serum 25-(OH)D levels, *p*= 0.62 (linear regression analysis), r^2^ = 0.005.

The 24 h urine protein had a negative association with serum 25-(OH)D, but it was not statistically significant (*p* = 0.62, linear regression analysis), with a correlation coefficient of −0.07 and r^2^ = 0.005 (as shown in Figure 1 and Table 3).

Complement C3, C4 levels and SLEDAI-2K, mex-SLEDAI scores were not statistically significant by linear regression analysis; however, we found a significant (*p* = 0.03) and positive relationship (correlation coefficient = 0.3) between age and 25-(OH)D levels (Table 3).

## 4. Discussion

We observed a high proportion of patients with low levels of 25-(OH)D, where only 13.2% of patients had sufficient levels of 25-(OH)D and 86.8% had low levels, of which 34.4% had deficiency (<10 ng/mL) and 52.4% insufficiency (10–30 ng/mL). Despite not having enough studies in Mexican patients, we found similar data in a study carried out in Mexico in 2017, with a prevalence of 25-(OH)D levels less than 30 ng/mL of 91.9% [17]. Studies in other populations have shown similar prevalence, which may vary depending on factors such as geographic, genetic, and environmental factors [12,13]. While we achieved comparable results, it is crucial to emphasize that our findings are merely frequencies, given that our study was retrospective and not all patients with SLE admitted during the study period had a blood 25-(OH)D measurement taken upon hospital admission.

Regarding demographic characteristics (Table 1), we found that younger patients had lower levels of 25-(OH)D than older patients, with an average of 23 years for the deficiency group and 39 years for the insufficiency group (*p* = 0.04); this result is consistent with what we found in the linear regression analysis between 25-(OH)D levels and age, where we found a significant (*p* = 0.03) and positive relationship (correlation coefficient = 0.3) between age and 25-(OH)D levels. In Bangladesh, Saha M et al. found similar data in their study that aimed to determine risk factors associated with low 25-(OH)D levels in patients with SLE, observing that younger patients had lower 25-(OH)D levels than older patients [38]. Although some studies indicated a correlation between low 25-(OH)D levels and elderly patients [39], others found no such relationship [17], the inconsistent findings may be ascribed to the restricted sample size of our investigation. Alternatively, the discrepancies could be attributed to other factors. The observed correlation between age and serum 25-(OH)D levels may be indicative of other underlying factors or clinical characteristics specific to the population under study. These could include seasonal variations in sampling, lifestyle factors, geographical origin, and other potential confounding variables [26]. We did not find a relationship with other variables among the demographic characteristics analyzed as possible risk factors associated with hypovitaminosis D, such as duration of the disease, 25-(OH)D supplementation, previous steroid consumption, use of immunomodulators, and body mass index, similar to what was reported in other studies [17] and contrary to the findings of Guan SY et al. [14], which indicated that patients lacking vitamin D supplementation exhibited reduced serum 25-(OH)D levels.

Reduced levels of 25-(OH)D 2343 were hypothesized to correspond with diminished levels of complement C3 and C4, together with disease activity [15,40]. Our investigation revealed reduced levels of complement C3 in 75% of patients within the deficiency cohort and 65% in the insufficiency cohort. Furthermore, reduced levels of complement C4 were noted in 50% of patients within the deficiency cohort and 56% within the insufficiency cohort. Although most patients displayed diminished levels of complement C3, no significant connection was observed between low serum levels of complement (C3 and C4) and serum levels of 25-(OH)D. Athanassiou et al. [39] observed that diminished complement levels correlated with reduced 25-(OH)D levels in individuals with SLE. Nonetheless, our analysis identified a positive association between serum levels of complement C3 and C4 and serum levels of 25-(OH)D; however, this link was weak and statistically insignificant. Unlike the research by Athanassiou et al., which compared complement levels in SLE patients with low 25-(OH)D levels to a control group with normal levels, our work examined serum complement levels in two SLE cohorts with varying serum 25-(OH)D levels. The results suggested that patients with diminished complement levels may display an increased prevalence of variations in serum 25-(OH)D levels. Nonetheless, further experimental data are necessary to validate the correlation between proportional reductions in blood 25-(OH)D levels and serum [41].

Our results showed a significant correlation between reduced levels of 25-(OH)D and elevated 24 h urine protein (*p* = 0.006). Nonetheless, the linear regression analysis indicated a weak and statistically insignificant negative correlation between 25-(OH)D levels and 24 h urine protein, implying that elevated protein levels are noted at diminished 25-(OH)D concentrations. Other cohorts of patients with systemic lupus erythematosus have documented observations regarding the correlation between diminished serum levels of 25-hydroxyvitamin D and renal impairment. Athanassiou et al. [39] identified persistent proteinuria (>0.5 g/24 h) as indicative of active disease and established a correlation between reduced levels of 25-(OH)D and renal involvement (*p* = 0.04), suggesting that renal disease may signify insufficient 25-(OH)D levels (<10 ng/mL). Additional evidence corroborating this finding encompass the expression of the 25-(OH)D receptor (VDR), which has been examined in kidney biopsy specimens, revealing that VDR expression was decreased in kidney biopsy samples from patients with lupus nephritis [42].

Currently, there are different scales for measuring SLE activity; in our study, we used the SLEDAI-2K and the mex-SLEDAI [36] (which is a modified and validated scale for measuring disease activity in Mexican patients). No significant variations in disease activity were seen between the 25-(OH)D deficient group and the vitamin D insufficiency group. We observed no significant differences in total SLEDAI-2K scores between the two groups. However, in the cohort with vitamin D deficiency, total SLEDAI-2K scores were significantly elevated when categorized into low-moderate and severe groups, in contrast to patients with vitamin D insufficiency. This suggested that a larger sample size may reveal significant differences pertaining to disease activity. The scores of both indices exhibited a negative connection with 25-(OH)D levels; specifically, greater SLEDAI-2K and mex-SLEDAI scores corresponded to lower vitamin D levels in the regression analysis. However, the link was weak and not statistically significant. Ruiz-Irastorza et al. [43] found analogous findings, after analyzing 92 individuals with SLE and finding no correlation between 25-(OH)D levels and the SLEDAI score, consistent with prior research [17]. Conversely, Amital et al. [40] indicated that 25-(OH)D had an inverse relationship with SLE activity in a cohort of 378 SLE patients from Europe and Israel; still, this connection was weak (r = −0.12, *p* = 0.018). Athanassiou et al. [39] noted a negative correlation between the SLEDAI-2K index score and serum 25-(OH)D levels (*p* < 0.001, r^2^ = 0.327). The inconsistency of these findings highlights the need for larger-scale clinical studies in populations from different countries that could help to confirm or refute this association.

It is still not clear whether low serum concentrations of 25-(OH)D are cofactors in the immunological disorders present in SLE or, on the contrary, the decreased sun exposure for photoprotection and kidney damage, among other factors associated with the disease, cause a further decrease in 25-(OH)D levels [21,44].

This study has certain limitations that must be acknowledged when evaluating the results. The patient sample in this study was limited, as numerous individuals with SLE admitted during the study period lacked serum 25-(OH)D level measurements, potentially rendering it unrepresentative of the broader SLE population in our country. A prospective design with multicenter patient inclusion would enable a more thorough characterization of the national population. Another limitation of this study is that the number of patients with SLE who had normal serum levels of 25-(OH)D (≥30 ng/mL) was very small (n = 8), and the medical records lacked the information necessary to make a comparison that could provide results with adequate statistical support. Another limitation to consider is that although the method used to measure 25-(OH)D is a precise method and even more sensitive than ELISA, it is not the method used in most articles. However, there are several articles reporting the equivalence of the results of different variables measured in serum, including 25-(OH)D [45,46]. Finally, in this exploratory study, we did not take into account the seasonal variation in serum 25-(OH)D levels [26], which could affect the results obtained, but we believe that the data obtained are relevant due to the limited information on this topic in the Mexican population and the exploratory nature of this study, which aims to allow future prospective controlled studies in which a greater number of variables can be controlled to allow greater statistical certainty. Despite these limitations, we consider our results important for adding to the available evidence on this important topic, which could have a great impact on the management and prognosis of SLE [14,31,40].

Despite the mixed and occasionally contradictory evidence regarding the efficacy of vitamin D supplementation in individuals with 25-(OH)D deficiency or insufficiency, it has been proposed that due to the common and extended use of corticosteroids in patients with SLE and the high incidence of low 25-(OH)D levels in this demographic, supplementation may be especially advantageous and could potentially decrease the corticosteroid dosages required for these patients [47,48].

## 5. Conclusions

This study found that 87% of individuals with SLE exhibited low serum 25-(OH)D levels. Patients with 25-(OH)D deficiency were significantly younger and demonstrated higher 24 h urine protein levels in comparison to those with 25-(OH)D insufficiency alone.

The findings enhance the current international literature regarding the increased prevalence of abnormal serum 25-(OH)D levels in patients with SLE. The regression analysis suggests a potential association among certain disease activity factors, age, and 25-(OH)D levels, warranting additional examination in subsequent research with a larger sample size.

## Figures and Tables

**Table 1:** Comparison of the demographic characteristics and clinical history of patients with SLE and 25-(OH)D deficiency or insufficiency.

		25-(OH)D Deficiency (n = 21)		25-(OH)D Insufficiency (n = 32)		OR (CI 95%)	*p*
		Median	IQR	Median	IQR		
Age		23	15	39	26	-	0.04
Years with diagnosis of SLE		1	4.9	1.5	5.5	-	0.44
		No.	%	No.	%		
Sex	Female	20	96	26	81	4.6 (1–55)	0.14
	Male	1	4	6	19		
BMI	Normal	15	71	18	56	1.9 (1–7)	0.26
	Sobrepeso-Obesidad	6	29	14	44		
Diagnosis	SLE	17	81	21	66	2.2 (1–7)	0.23
	SLE + Another auntoimmune disease	4	19	11	34		
Vitamin D supplementation	Yes	15	71	17	53	2.2 (1–7)	0.18
	No	6	29	15	47		
History of use of Steroids	Yes	15	71	25	78	0.7 (0.2–2.4)	0.58
	No	6	29	7	22		
History of use of immunomodulators	Yes	19	90	28	87	1.4	0.74
	No	2	10	4	13	(0.3–7.6)	

25-(OH)D = 25-hydroxyvitamin D, BMI = body mass index, CI = confidence interval, IQR = interquartile range, SLE = systemic lupus erythematosus.

**Table 2:** Comparison of the clinical and laboratory characteristics of patients with SLE and the presence of 25-(OH)D deficiency or insufficiency.

		25-(OH)D Deficiency (n = 21)		25-(OH)D Insufficiency (n = 32)			
		Median	IQR	Median	IQR	OR (CI 95%)	*p*
**Hemoglobin (g/dL)**		9.9	3.9	10.3	5.3	-	0.26
**Platelets (×10^9^/L)**		128	125	197	229	-	0.52
**Leukocytes (×10^9^/L)**		5.2	6.1	6.5	7.1	-	0.07
**Lymphocytes (×10^9^/L)**		0.7	1	1.4	1.2	-	0.12
**Creatinine (g/dL)**		1	2.4	0.7	0.5	-	0.18
**ESR (mL/h)**		45	47	40	36	-	0.45
**CRP (mg/dL)**		2.3	6.2	3	2.3	-	0.16
**24-h urine protein (g/24 h)**		1.8	1.3	1.1	0.8	-	0.006
		n	%	n	%		
**Complement C3 level**	Normal	5	25	11	34	0.6 (0.2–2.1)	0.48
	Low	15	75	21	66		
**Complement C4 level**	Normal	10	50	14	44	1.3 (0.4–4.2)	0.66
	Low	10	50	18	56		
**SLEDAI-2K score**	Low-Moderate	15	71	19	59	1.7 (0.5–5.6)	0.37
	Severe	6	29	13	41		
**mex-SLEDAI score**	Active	17	81	21	66	2.2 (0.6–7.2)	0.23
	Inactive	4	19	11	34		

25-(OH)D = 25-hydroxyvitamin D, BMI = body mass index, CI = confidence interval, CRP = C-reactive protein, ESR = erythrocyte sedimentation rate, IQR = interquartile range, mex-SLEDAI = Mexican-Systemic Lupus Erythematosus Disease Activity Index score, SLE = systemic lupus, SLEDAI-2K = Systemic Lupus Erythematosus Disease Activity Index 2000 score.

**Table 3:** Results of linear regression analysis for variables associated with SLE activity and linear regression analysis for age.

	Correlation Coefficient	r^2^	*p*
**24-h urine protein**	−0.07	0.005	0.62
**Complement C3**	0.22	0.05	0.12
**Complement C4**	0.1	0.01	0.47
**SLEDAI-2k**	−0.14	0.02	0.32
**mex-SLEDAI**	−0.17	0.03	0.24
**Age**	0.3	0.09	0.03

mex-SLEDAI = Mexican Systemic Lupus Erythematosus Disease Activity Index score, SLEDAI-2K = Systemic Lupus Erythematosus Disease Activity Index 2000 score.

## Data Availability

The data presented in this study are available upon request from the corresponding author due to privacy reasons.
